# DCZ3301, a novel cytotoxic agent, inhibits proliferation in diffuse large B-cell lymphoma via the STAT3 pathway

**DOI:** 10.1038/cddis.2017.472

**Published:** 2017-10-12

**Authors:** Xi Sun, Bo Li, Bingqian Xie, Zhijian Xu, Gaomei Chang, Yi Tao, Yong Zhang, Shuaikang Chang, Yingcong Wang, Dandan Yu, Yongsheng Xie, Tingye Li, Houcai Wang, Gege Chen, Liangning Hu, Jun Hou, Yiwen Zhang, Wenqin Xiao, Lu Gao, Jumei Shi, Weiliang Zhu

**Affiliations:** 1 Department of Hematology, Shanghai Tenth People’s Hospital, Tongji University Cancer Center, Tongji University School of Medicine, Shanghai 200072, China; 2 CAS Key Laboratory of Receptor Research, Drug Discovery and Design Center, Shanghai Institute of Materia Medica, Chinese Academy of Sciences, Shanghai 201203, China; 3 Nanjing Medical University School of Clinical Medicine, Nanjing 210000, China

## Abstract

Diffuse large B-cell lymphoma (DLBCL) is the most common type of lymphoma in adults, characterized by a rapidly increasing painless mass. A novel compound, DCZ3301, was synthesized that exerted direct cytotoxicity against DLBCL cell lines. The effects of DCZ3301 on DLBCL cells *in vitro* and *in vivo* and the associated mechanisms were investigated. DCZ3301 inhibited the viability of DLBCL cell lines, even in the presence of protumorigenesis cytokines. Additionally, the compound induced apoptosis and cell cycle arrest at the G2/M phase by reducing mitochondrial membrane potential. DCZ3301 exerted an antitumor effect through modulation of Akt, extracellular signal-regulated kinases 1/2 (ERK1/2) and janus kinase 2 (JAK2)/signal transducer and activator of transcription 3 (STAT3) signaling pathways. Furthermore, DCZ3301 downregulates STAT3 phosphorylation by inhibiting Lck/Yes-related novel protein tyrosine kinase (Lyn) activation in DLBCL. A synergistic cytotoxic effect on DLBCL cells was observed upon combination of DCZ3301 with panobinostat. *In vivo*, intraperitoneal injection of xenograft mice with DCZ3301 resulted in reduced tumor volume. Our preliminary results collectively support the utility of the small-molecule inhibitor DCZ3301 as an effective novel therapeutic option for DLBCL that requires further clinical evaluation.

Diffuse large B-cell lymphoma (DLCBL) is the most common subtype of lymphoid cancer, accounting for ~30% of all lymphoma cases.^1, 2^ DLBCL is a clinically and genetically heterogeneous lymphoid malignancy mainly characterized by two major molecular subtypes representing different stages of B-cell lymphoid differentiation based on gene expression profiling, specifically activated B-cell-like and germinal center B-cell-like.^3, 4^ Although the current standard chemotherapy regimen of rituximab plus cyclophosphamide, vincristine, doxorubicin and prednisone (R-CHOP) for DLCBL patients can enhance response rates (RR) and prolong the survival of patients, >30% of patients still fail to respond or show relapse with resistant disease.^5, 6^ Therefore, the development of novel drugs or therapies that can be effectively applied to improve the outcomes of DLBCL patients is an essential medical need.

The phosphatidylinositol 3-kinase (PI3K)/Akt/mTOR signal pathway is one of the most prominent pathways against malignant lymphoma, and PI3K/Akt inhibitors have been shown to be effective in DLBCL.^7^ Extracellular signal-regulated kinase 1/2 (ERK1/2) is a member of the mitogen-activated protein kinase family that regulates cell proliferation and survival associated with genomic instability.^8^ The ERK1/2 pathway is activated during apoptotic cell death of DLBCL.^9^ Signal transducer and activator of transcription 3 (STAT3) activated in B-cell lymphoma patients present an attractive target for therapeutic development with the potential of inhibiting cancer cell growth.^10, 11^ Research on a large sample of DLBCL patients treated with R-CHOP showed that activation of the STAT3 signaling pathway is related to shorter survival.^12^ Furthermore, targeting the STAT3 pathway presents a potential approach to reverse CHOP resistance in patients with DLBCL.^13^ Lck/Yes-related novel protein tyrosine kinase (Lyn), belonging to Src tyrosine kinase family, is expressed preferentially in the membrane of B cells and other hematopoietic cells rather than T cells.^14, 15^ The activation of Lyn, which leads to STAT3 phosphorylation,^16^ has an important role in B-cell activation in lymphoma cells.^17, 18, 19^

In the present study, we showed that DCZ3301, a newly synthesized aryl-guanidino agent, exerts an antitumor effect via inhibiting proliferation of DLBCL cells, both *in vitro* and *in vivo*. Moreover, DCZ3301 induced apoptosis and cell cycle arrest by regulating Akt, ERK1/2 and STAT3 pathways in DLBCL cells without exerting cytotoxicity in normal cells. The antitumor activity of DCZ3301 in a mouse xenograft model and the molecular mechanisms underlying DCZ3301-mediated induction of apoptosis were further investigated. Our findings suggest that DCZ3301 can be effectively applied as a novel potential therapeutic regimen for DLBCL.

## Results

### DCZ3301 inhibits DLBCL cell proliferation

**Figure 1:**
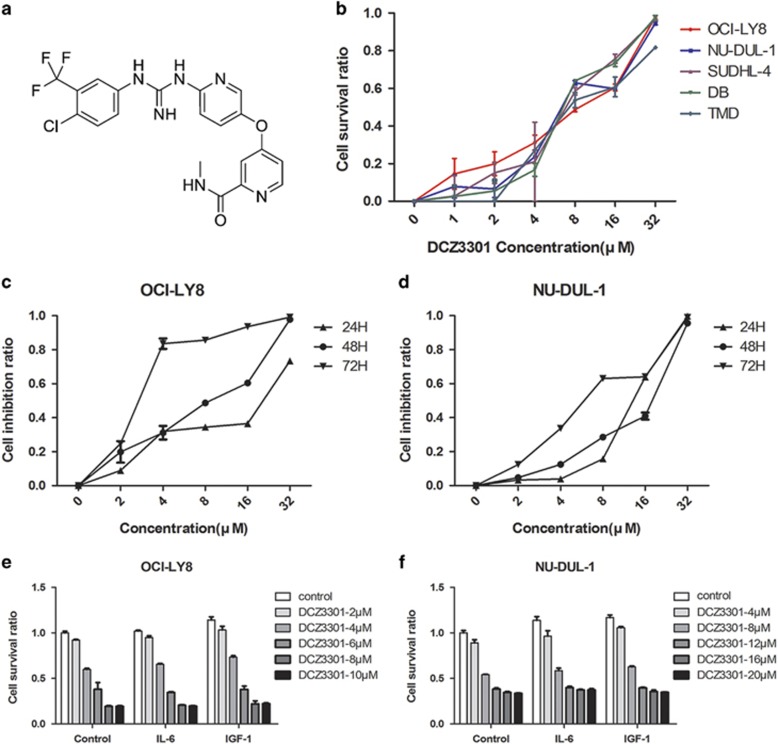
DCZ3301 inhibits the proliferation of the DLBCL cell lines. (**a**) The chemical structure of DCZ3301. (**b**) DLBCL cell lines (OCI-LY8, NU-DUL-1 SUDHL-4, DB and TMD8) were treated with DCZ3301 (1, 2, 4, 8, 16 and 32 *μ*M) in 96-well plates for 48 h. (**c**) OCI-LY8 and (**d**) NU-DUL-1 cell lines were treated with DCZ3301 (1, 2, 4, 8, 16 and 32 *μ*M) in 96-well plates for 24, 48 and 72 h. (**e**) OCI-LY8 and (**f**) NU-DUL-1 cells were treated with the indicated concentrations of DCZ3301 for 48 h in the presence or absence of either 50 nM IL-6 or 25 nM IGF-1 for 24, 48 and 72 h. The processed cells were followed by assessment for cell proliferation using CCK-8

As shown in Figure 1a, DCZ3301 is a newly synthesized compound with a molecular weight of 464.0 Da. To investigate the efficacy of DCZ3301 in DLBCL, OCI-LY8, NU-DUL-1 SUDHL-4, DB and TMD8 cells were treated with DCZ3301 at concentrations of 1, 2, 4, 8, 16 and 32 *μ*M. Proliferation of DLBCL cells was detected using the Cell Counting Kit-8 (CCK-8) assay. Treatment with DCZ3301 for 48 h resulted in a dose-dependent decrease in DLBCL cell proliferation (Figure 1b). The calculated half-maximal inhibitory concentration (IC50) values were as follows: 7.1 (OCI-LY8), 9.7 (NU-DUL-1), 6.67 (SUDHL-4), 8.04 (DB), and 9.66 *μ*M (TMD8). DCZ3301 significantly inhibited the proliferation of DLBCL cells (OCI-LY8 and NU-DUL-1) in a time-dependent manner (Figures 1c and d). We further explored DLCBL proliferation by treatment with DCZ3301 in the presence or absence of interleukin-6 (IL-6) and insulin-like growth factor-1 (IGF-1), given that cytokines have an important role in lymphoma growth and survival. Although both IL-6 and IGF-1 alone can stimulate DLBCL cell growth, DCZ3301-induced growth inhibition was not influenced by these cytokines (Figures 1e and f).

### DCZ3301 induces apoptosis in DLBCL cells

**Figure 2:**
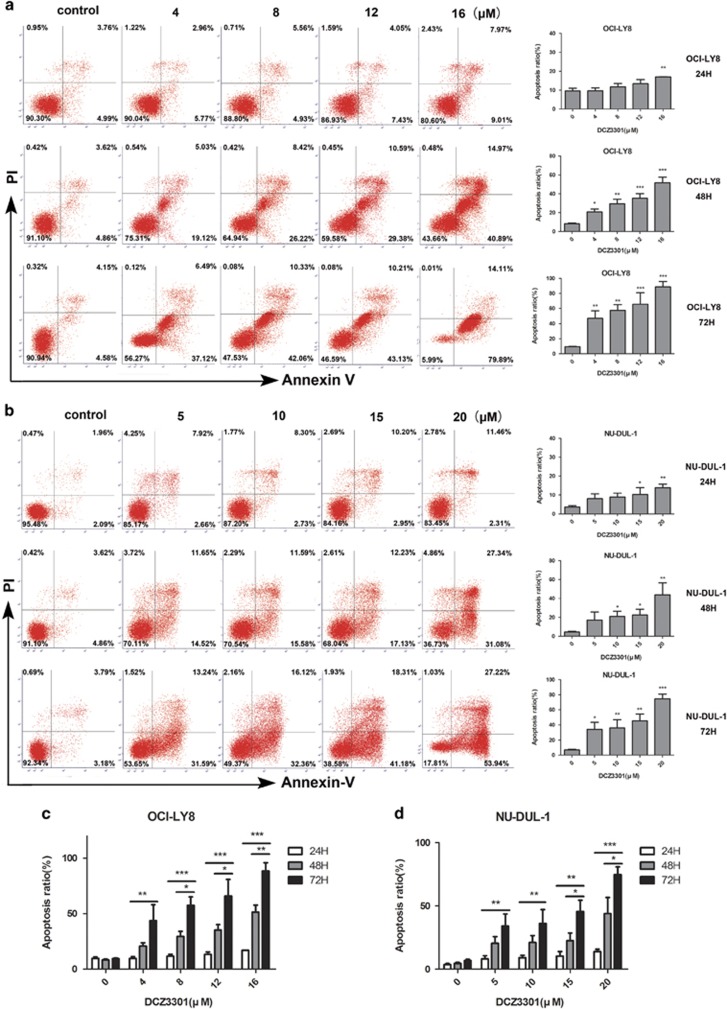
DCZ3301 induces apoptosis in DLBCL cells. (**a**) OCI-LY8 cells were treated with DCZ3301 (4, 8, 12 and 16 *μ*M) for 24, 48 and 72 h while (**b**) NU-DUL-1 cells were treated with DCZ3301 (5, 10, 15 and 20 *μ*M) for 24, 48 and 72 h, double-stained with Annexin-V/propidium iodide (PI) and analyzed by flow cytometry. Columns show the percentage of Annexin-V positive cells from three independent experiments, data shown as the means±S.D. (*P<0.05; **P<0.01, ***P<0.001). (**c**) Analysis of OCI-LY8 and (D) NU-DUL-1 cells treated with certain concentrations of DCZ3301 at different time points (24, 48 and 72 h) were performed by Annexin-V/PI. The ratio of apoptotic cells is shown as means±S.D. (*P<0.05, **P<0.01, ***P<0.001)

The apoptotic effect of DCZ3301 on DLBCL cells was examined via Annexin-V/propidium iodide (PI) double staining. Compared with the control group, apoptosis was distinctly induced in a time- and dose-dependent manner in OCI-LY8 and NU-DUL-1 cells by DCZ3301 (Figures 2a–d). These results were consistent with data obtained from the CCK-8 assay.

### DCZ3301 triggers a decrease in mitochondrial membrane potential

**Figure 3:**
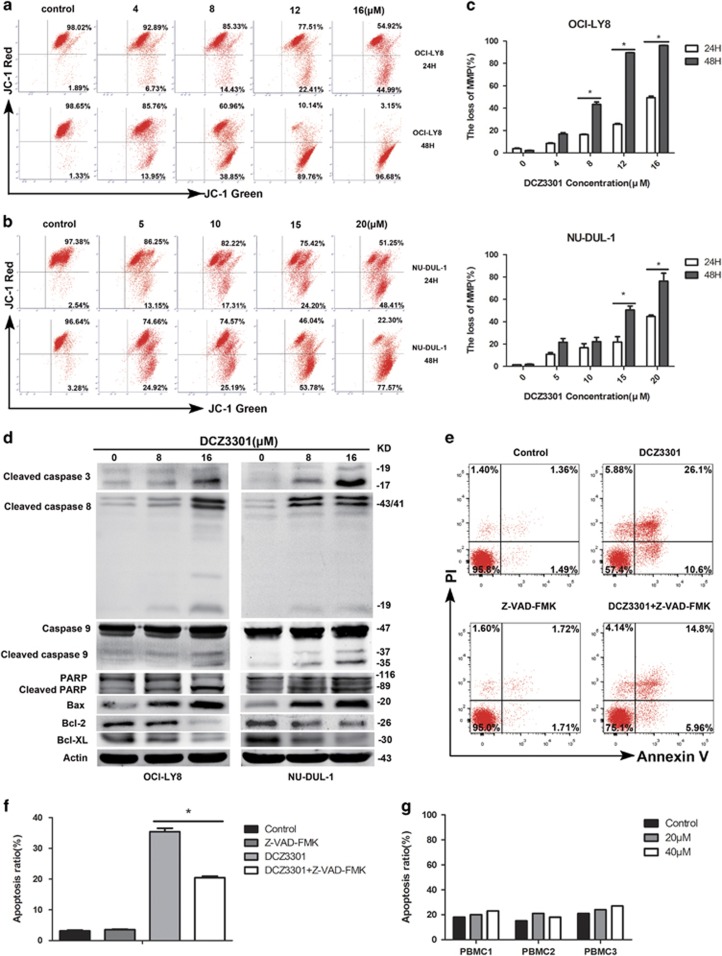
DCZ3301 induces MMP decreases and enhances caspase activation. (**a**) OCI-LY8 cells treated with DCZ3301 (4, 8, 12 and 16 *μ*M) and (**b**) NU-DUL-1 cells treated with DCZ3301 (5, 10, 15 and 20 *μ*M) for 24 and 48 h were stained with JC-1 to examine the level of MMP by flow cytometry. (**c**) Assessment of OCI-LY8 and NU-DUL-1 cells treated with DCZ3301 by JC-1 showed change in MMP. Columns show the loss of MMP represented as means±S.D. (*P<0.05). (**d**) Western blotting analysis was performed to explore the protein levels of caspase family, including cleaved caspase-3, capase-8 and caspase-9, PARP and B-cell lymphoma (Bcl) family, such as Bcl-2, Bcl-xL and Bax. (**e**) NU-DUL-1 cells incubated with or without pan-caspase inhibitor Z-VAD-FMK were both treated with 15 *μ*M DCZ3301 for 48 h, followed by Annexin-V/propidium iodide (PI) analysis on flow cytometry. (**f**) Columns show the percentage of Annexin-V positive cells from three independent experiments, which are shown as means±S.D. (*P<0.05). (**g**) Normal PBMCs from three healthy volunteers treated with different concentrations of DCZ3301 (0, 20 and 40 *μ*M) for 48 h were stained with Annexin-V/PI and analyzed by flow cytometry; the ratio of apoptotic cells shown as mean±S.D.

Mitochondrial membrane potential (MMP), which participates in mitochondrial oxidative phosphorylation, is decreased prior to early pathological changes in the intrinsic pathway of apoptosis.^20^ To explore whether DCZ3301 affects mitochondrial depolarization, we used the JC-1 MMP detection kit to evaluate loss of MMP. Notably, relative to the control group, DCZ3301 treatment led to loss of MMP, in turn, activating the intrinsic apoptosis pathway, as shown in Figures 3a–c. Additionally, MMP reduction by DCZ3301 occurred in a time- and concentration-dependent manner.

### DCZ3301 treatment enhances caspase activation

To further clarify the molecular mechanism underlying DCZ3301-induced apoptosis in DCBCL cells, we detected the presence of cleaved caspase-3, caspase-8, caspase-9, poly ADP-ribose polymerase (PARP) and mitochondrial apoptotic pathway proteins (Bcl-2 family) via western blotting. As shown in Figure 3d, treatment of OCI-LY8 and NU-DUL-1 cells (8 or 16 *μ*M) with DCZ3301 caused dose-dependent increase in the cleaved forms of caspase-3, caspase-8 and caspase-9 as well as PARP, indicating that DCZ3301 induces apoptosis in DLBCL cells through both extrinsic and intrinsic pathways. Furthermore, Z-VAD-FMK, a pan-caspase inhibitor, suppressed apoptosis of DCZ3301-treated NU-DUL (Figures 3e and f) and OCI-LY8 cells (data not shown). Downregulation of Bcl-2 and Bcl-xL and, conversely, upregulation of Bax confirmed disruption of mitochondrial integrity by treating DCZ3301 at the proteomic level. In addition, DCZ3301 exerted no significant effect on normal peripheral blood mononuclear cells (PBMCs), even at a concentration of 40 *μ*M, compared with the control group (Figure 3g), supporting its safety for use as a therapeutic agent for DLBCL.

### DCZ3301 arrests the cell cycle at the G2/M phase in DLBCL

**Figure 4:**
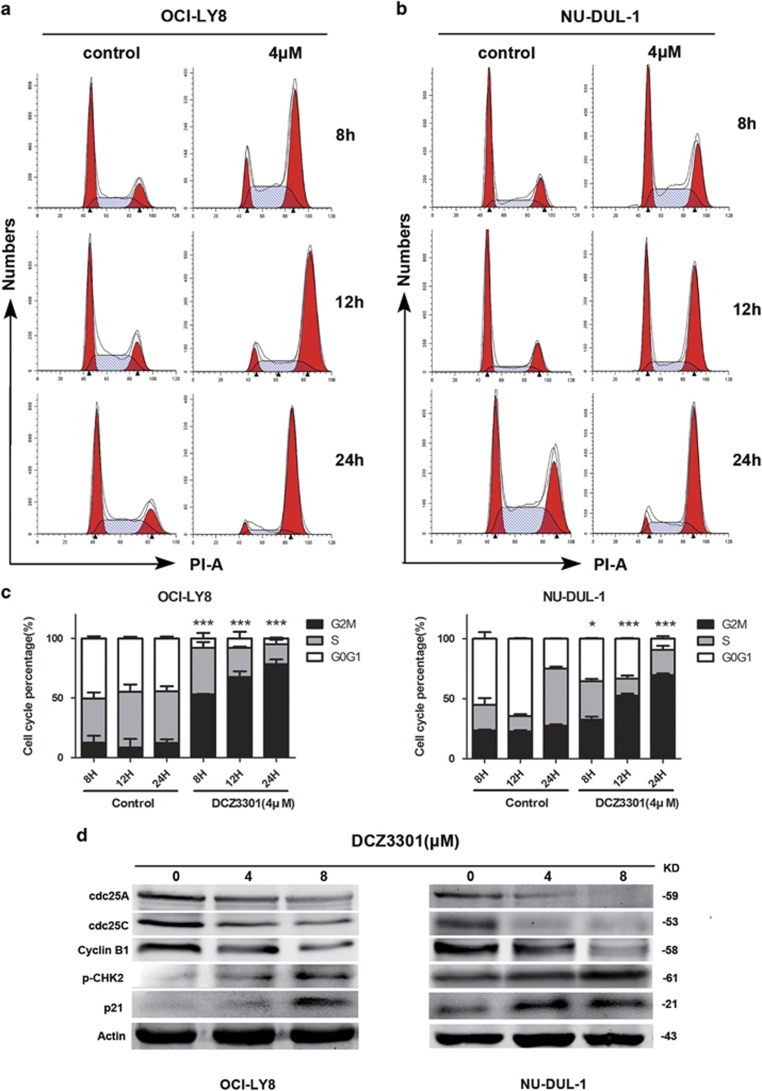
DCZ3301 arrests the cell cycle at G2/M phase in DLBCL cells. (**a**) OCI-LY8 cells and (**b**) NU-DUL-1 cells were treated with DCZ3301 (4 *μ*M) for 8, 12 and 24 h, stained with PI and analyzed on flow cytometry. (**c**) The percentage of G0/G1, S and G2/M phase cells after control or DCZ3301 treatment at different time points (8, 12 and 24 h). Data are shown as mean±S.D. (*n*=3, *P<0.05; **P<0.01; ***P<0.001). (**d**) OCI-LY8 and NU-DUL-1 cells were treated with DCZ3301 (4 and 8 *μ*M) for 24 h.The protein levels of p-CHK2, cyclinB1, cdc25A, cdc25C, p21 and Actin were assessed by western blotting

In view of the finding that DCZ3301 induces apoptosis in DLBCL cells, we assessed its effect on the cell cycle, which is also associated with proliferation, using flow cytometry. After treatment of OCI-LY8 and NU-DUL-1 cells with DCZ3301, the percentage of cells in the G2/M phase accumulated significantly in a time-dependent manner in both cell lines (Figures 4a–c). To further elucidate the molecular mechanisms underlying DCZ3301 induction of G2/M phase arrest, we examined the protein levels of phospho-checkpoint kinase2 (p-CHK2), cell division cycle 25A (cdc25A), cdc25C, cyclinB1 and p21 via western blotting analysis. As shown in Figure 4d, DCZ3301 caused an increase in p21 and p-CHK2 protein expression. Meanwhile, the protein levels of cdc25A, cdc25C and cyclinB1, which has an important role in G2/M phase arrest, were decreased in DCZ3301-treated DLBCL cells.

### Akt, ERK 1/2 and JAK2/STAT3 pathways are regulated in DCZ3301-induced apoptosis

**Figure 5:**
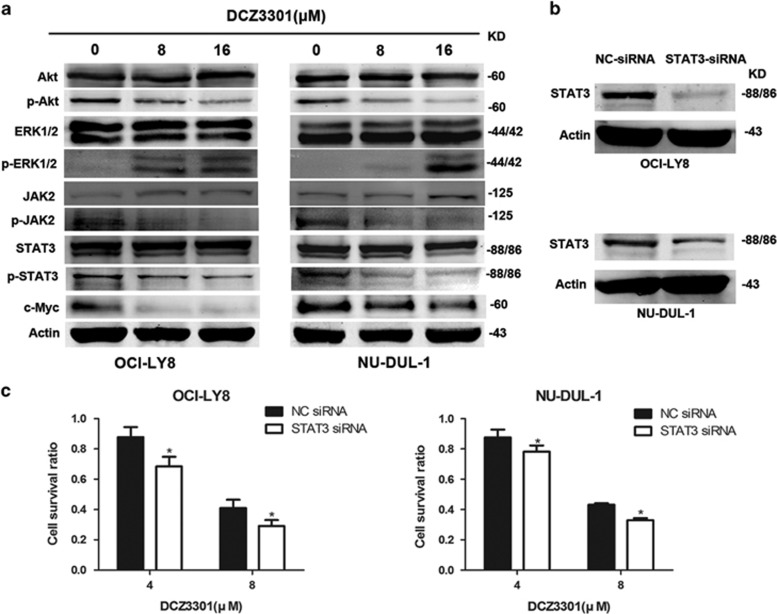
DCZ3301 regulates the signaling pathways of Akt, ERK 1/2 and STAT3. (**a**) OCI-LY8 and NU-DUL-1 cells were treated with the indicated concentrations of DCZ3301 (8 and 16 *μ*M) for 48 h. Western blotting analysis was performed to examine the protein levels of Akt, phospho-Akt, ERK1/2, phospho-ERK1/2, janus kinase 2 (JAK2), phospho-JAK2, STAT3, phospho-STAT3, c-Myc and Actin. (**b**) OCI-LY8 and NU-DUL-1 cells were both transfected with STAT3 siRNA and negative-control siRNA, respectively. The protein levels of STAT3 and Actin were analyzed by western blotting. (**c**) The transfected DLBCL cells were treated with 4 and 8 *μ*M DCZ3301 for 48 h, following cell proliferation assessment using CCK-8

We further assessed the expression patterns of molecules involved in several classic pathways via western blotting to determine the mechanism underlying DCZ3301-induced apoptosis. Upregulation of phosphorylated ERK1/2 and downregulation of phosphorylated Akt were enhanced in OCI-LY8 and NU-DUL-1 cells treated with DCZ3301 (Figure 5a). Additionally, the JAK2/STAT3 pathway was suppressed on account of decreased phosphorylated STAT3 and JAK2. The c-Myc oncogene, a prognostic factor of DLBCL,^21^ was also reduced by DCZ3301 in a dose-dependent manner. To explore the pathway involved in the process of DCZ3301 treatment, knockdown of STAT3 was performed via siRNA in OCI-LY8 and NU-DUL-1 cells (Figure 5b). The efficacy of DCZ3301 was enhanced in this group, compared with that of the negative control siRNA group (Figure 5c), suggesting that STAT3 downregulation has a functional role in DCZ3301-induced apoptosis in DLBCL cells. Together, we found that DCZ3301 induces cell apoptosis in DLBCL cells by regulating JAK2/STAT3, Akt and ERK1/2 signal pathways.

### STAT3 phosphorylation is inhibited by Lyn activation in DLBCL

**Figure 6:**
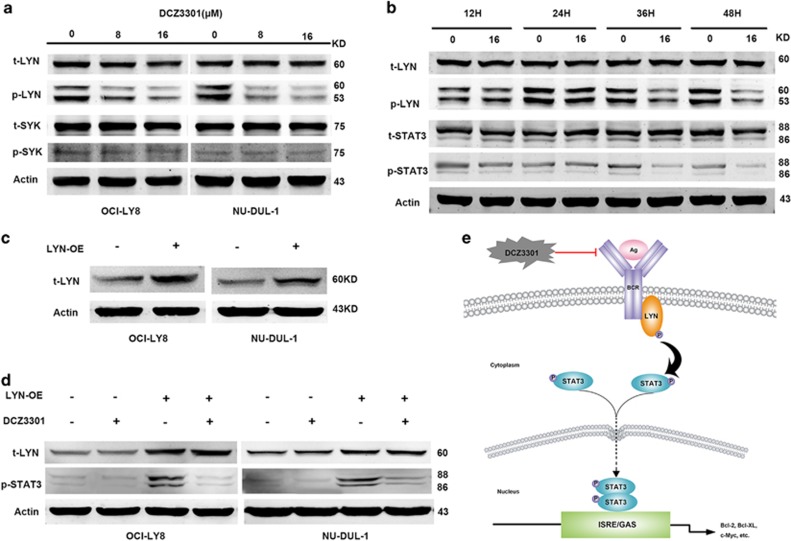
STAT3 phosphorylation is inhibited by Lyn activation in DLBCL. (**a**) OCI-LY8 and NU-DUL-1 cells were treated with DCZ3301 (8 and 16 *μ*M) for 48 h and assessed the protein levels of Lyn, phospho-Lyn, Syk, phospho-Syk and Actin by performing western blotting analysis. (**b**) The protein levels of Lyn, phospho-Lyn, STAT3, phospho-STAT3 and Actin in OCI-LY8 cells treated with DCZ3301 (16 *μ*M) for 12, 24, 36 and 48 h were examined by western blotting analysis. (**c**) OCI-LY8 and NU-DUL-1 cells were transfected with a plasmid that overexpresses oncogene Lyn and an empty vector, respectively. The protein levels of Lyn and Actin were analyzed by western blotting. (**d**) The transfected OCI-LY8 and NU-DUL-1 cells were treated with DCZ3301 (0 and 16 *μ*M) for 24 h and the protein levels of Lyn, phospho-STAT3 and Actin were examined by western blotting analysis. (**e**) The signal transduction pathway involving in STAT3 activation that DCZ3301 induced in cell apoptosis of DLBCL cells

Whereas STAT3 inactivation has an important role in DCZ3301-induced apoptosis, we investigated the oncogene upstream that induces STAT3 phosphorylation, such as tyrosine kinase Lyn and Syk.^16, 22^ As shown in Figure 6a, we found that the phosphorylation (Y507) of tyrosine kinase Lyn, rather than Syk, was downregulated by DCZ3301 treatment in DLBCL cells. Furthermore, our results demonstrated that the decrease of phosphorylated Lyn was enhanced with the passing of time (Figure 6b), suggesting that DCZ3301 inhibits the phosphorylation of Lyn in both time- and dose-dependent manner in DLBCL cells. On the other hand, we surprisingly found that DCZ3301 decreases the tyrosine phosphorylation of STAT3 in a manner that is very similar to Lyn (Figure 6b), suggesting that Lyn and STAT3 phosphorylation are suppressed synchronously after DCZ3301 treatment at the protein level. To explore whether the interaction between Lyn and STAT3 involved in the antilymphoma effect induced by DCZ3301, we overexpressed Lyn in DLBCL cell lines by transfecting a recombination plasmid (Figure 6c) and treated with or without DCZ3301. Our current data showed that Lyn-overexpressive (Lyn-OE) cells exhibit much higher level of phosphorylated STAT3 than negative control cells (Figure 6d). In addition, the suppression of phosphorylated STAT3 was observed more in Lyn-OE cells than in negative control cells after DCZ3301 treatment for only 24 h (Figure 6d). Based on the collective findings, we propose that DCZ3301 promotes DLBCL cell apoptosis through modulating STAT3 signaling by inhibiting Lyn activation (Figure 6e).

### DCZ3301 inhibits tumor growth *in vivo*

**Figure 7:**
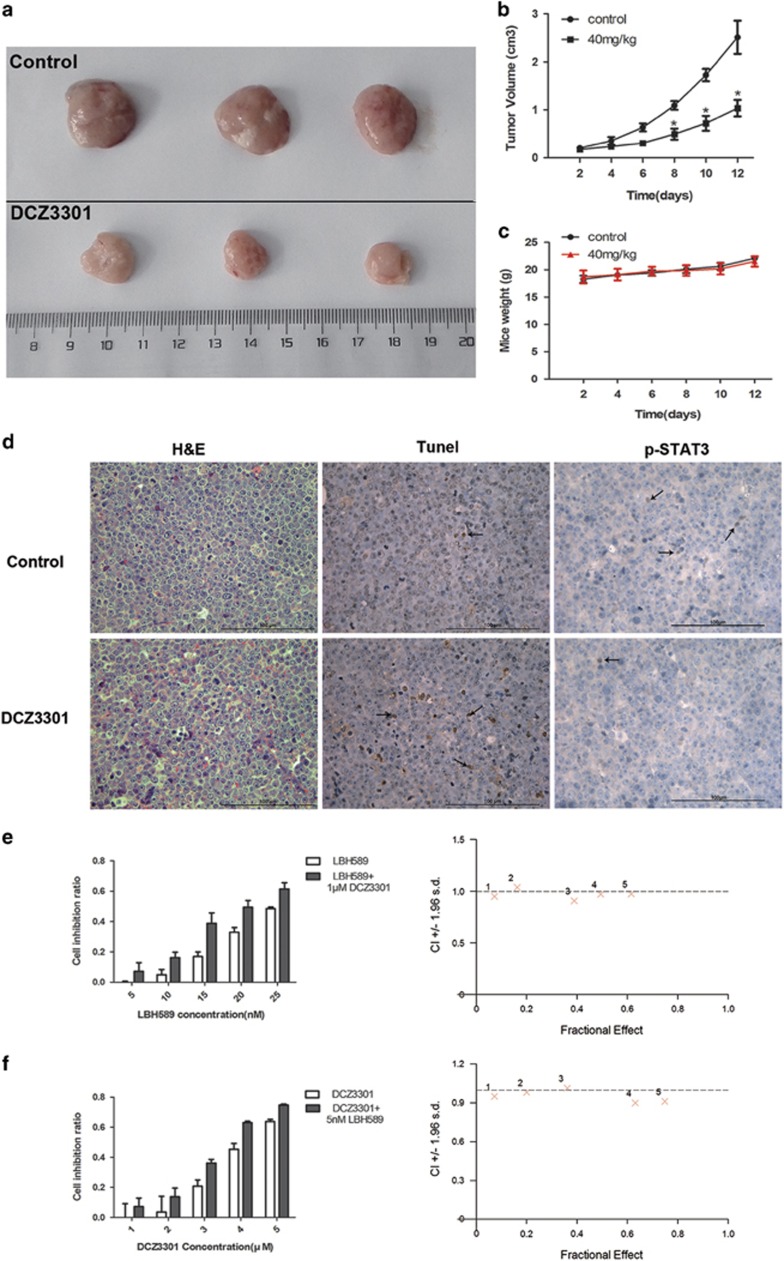
DCZ3301 inhibits the growth of tumor in a xenograft mouse model and synergizes with panobinostat in DLBCL cell lines. OCI-LY8 cells (2.0 × 10^6^) were subcutaneously injected into the flank of 6-week-old nude mice and, respectively, administered 5% DMSO and saline to the controlled group or DCZ3301 (40 mg/kg) to the drug-treated group for 12 days (*n*=3/group). (**a**) The tumor samples were collected and imaged using a digital camera. (**b**) The tumor volume was measured each day for 12 days (*P<0.05). (**c**) The weight of mice was measured each day for 12 days. (**d**) H&E, TUNEL and anti-phospho-STAT3 antibody staining of tumor tissues from controlled or DCZ3301-treated mice were pictured (original magnification: × 400). (**e**) OCI-LY8 cells were treated with panobinostat or panobinostat at a constant concentration of DCZ3301 or (**f**) were treated with DCZ3301 or DCZ3301 at a constant concentration of panobinostat and then the cell proliferation was detected using CCK-8 assay after culturing for 48 h. Data analyzed by the Calcusyn software shows the synergistic activity of DCZ3301 and panobinostat against DLBCL cell lines. Combination index *<*1 indicates synergy

We further investigated the therapeutic efficacy of DCZ3301 *in vivo* by establishing a nude mouse xenograft model. Specifically, OCI-LY8 cells were injected into 6-week-old male BALB/c nude mice. Once the tumor volume reached the appropriate size, mice were treated with 5% dimethyl sulfoxide (DMSO) and saline or DCZ3301 via intraperitoneal injection. During the experimental period, volume, weight and the general state of all mice were measured every day to determine the antitumor effect and lethal toxicity or other side effects following DCZ3301 treatment. Administration of DCZ3301 induced a significant decrease in tumor growth (Figures 7a and b) while mouse weight was not significantly different between the DCZ3301-treated, 5% DMSO and saline groups (Figure 7c). Cell necrosis was observed via hematoxylin and eosin (H&E) and TUNEL staining in tumors of the DCZ3301-treated group, compared with those of the control group (Figure 7d). In addition, the expression of phospho-STAT3 in the tumor tissues was downregulated by DCZ3301 treatment (Figure 7d). During the process of drug administration, microscopic observation of functional organs revealed no evidence of growth disorder or organ dysfunction (data not shown). Our findings support the safety and efficacy of DCZ3301 as a promising novel treatment for lymphoma.

### DCZ3301 acts synergistically with panobinostat in DLBCL cells

To ascertain whether DCZ3301 can be effectively used in combination therapy, we examined the proliferation of OCI-LY8 cells treated with increasing concentrations of DCZ3301 in conjunction with specific concentrations of panobinostat. Combination of DCZ3301 and panobinostat generally induced synergetic cytotoxicity in OCI-LY8 cells indicated by combination index *<*1.0 for the most part (Figures 7e and f). Combination of DCZ3301 with the histone deacetylase inhibitor vorinostat led to a weak synergistic effect (data not shown).

## Discussion

DLBCL is the most common lymphoma type in adults worldwide that presents a substantial clinical problem.^23^ Even following standard chemotherapy with R-CHOP, >30% patients undergo relapse/refractory issues.^5^ Effective chemotherapeutic agents are therefore urgently required to improve therapeutic outcomes.

DCZ3301, a novel aryl-guanidino compound synthesized in our laboratory, shows the activity in multiple cancer cell types, particularly hematological tumors. Here we investigated the antitumor effects of DCZ3301 on human DLBCL cells and the associated mechanisms. The inhibitory effects of DCZ3301 on DLBCL cell lines were induced through apoptosis as well as G2/M phase cell cycle arrest. Concomitantly, DCZ3301 exerted no significant toxicity in PBMCs. In a DLBCL xenograft mouse model, administration of DCZ3301 led to inhibition of neoplasm growth, consistent with *in vitro* data.

We further assessed inhibition of proliferation induced by DCZ3301 in a DLBCL cell model. Initially, a dose- and time-dependent cytotoxic effect was demonstrated in the human DLBCL cell lines OCI-LY8, NU-DUL-1, SUDHL-4, TMD8 and DB, with an approximate IC50 value of 8 *μ*M after DCZ3301 treatment for 48 h. Recent studies have reported that deficiency of IL-6 protects against B-cell lymphomagenesis^24^ while serous IL-6 levels are associated with prognosis of DLBCL.^25^ Moreover, downregulation of IGF-1 led to reduced proliferation of DLBCL cell lines through inhibition of the IGF-1 receptor.^23^ In the current study, IL-6- and IGF-1-cultured cells were significantly increased compared with the control group, indicating a role of these cytokines in promoting disease progression. Interestingly, however, DCZ3301-induced cell cytotoxicity was not significantly weakened in the presence of IL-6 and IGF-1, suggesting that DCZ3301 blocks the proliferation-associated pathways triggered by these cytokines.

DCZ3301 induced apoptosis in a dose- and time-dependent manner in the apoptosis study, consistent with data obtained with the CCK-8 assay. Experiments performed to establish whether the antitumor effect of DCZ3301 is exerted via a caspase-dependent apoptotic pathway revealed caspase activation, as evident from the increased presence of the cleaved forms of caspase-8, caspase-9, caspase-3 and PARP. The data indicate that DCZ3301 induces apoptosis in OCI-LY8 and NU-DUL-1 cells via both extrinsic and intrinsic apoptotic pathways. Caspase-8 and caspase-9 are the two key proteins, respectively, activated in the extrinsic and intrinsic apoptotic pathways, together resulting in PARP and caspase-3 cleavage occurring downstream of the entire apoptotic pathway.^26^ We further examined MMP, which is altered at the early stages of intrinsic apoptosis.^27^ As an indicator of mitochondrial membrane permeability during early apoptosis, MMP decrease leads to activation of caspase-9.^28^ JC-1 assessment demonstrated that DCZ3301 disrupts mitochondrial function by inducing mitochondrial depolarization during the process of intrinsic apoptosis. This inference was supported by mitochondrial protein analysis showing decreased Bcl-2 and Bcl-xL and concomitantly increased Bax in DCZ3301-treated DLBCL cells. Additionally, DCZ3301 exerted no significant cytotoxic effects on normal cells, supporting its safety of use.

As inhibition of cell proliferation is induced not only by cell apoptosis but also cell cycle arrest,^29^ we evaluated the effect of DCZ3301 on the cell cycle. DCZ3301 arrested cell cycle at the G2/M phase. CHK2 is an important protein kinase in the DNA damage response pathway that can directly regulate cdc25A and cdc25C proteins associated with cell cycle control.^30^ Downregulated cdc25A and cdc25C prevent cyclinB1 from combining to form the CDK1–cyclinB1 complex.^31, 32^ The cell cycle is arrested at the G2/M phase, and ultimately, apoptosis is promoted if CDK1-cyclinB1 kinase activity is inhibited. On the other hand, CHK2 can also promote p21 accumulation, which leads to G2/M arrest.^33^ Our results suggest that DCZ3301 induces DNA damage by inhibiting CDK1–cyclinB1 kinase activity through both p21 and cdc25A/cdc25C pathways in DLBCL.

We further investigated the molecular mechanisms underlying DCZ3301 lethality in DLBCL. PI3K/Akt/mTOR is an important pathway in DLBCL, considering that its inhibition results in antilymphoma activity both *in vivo* and *in vitro*, suggesting that Akt is a potential target for DLBCL treatment.^34, 35^ The ERK1/2 pathway also has a crucial role in cell proliferation, differentiation, apoptosis and migration, which has been verified in lymphoma cells.^36^ Data from the current study showed that DCZ3301 inhibits the Akt pathway while activating the ERK pathway. PI3K/Akt/mTOR and ERK are documented to be highly correlated with STAT3 signaling. The Akt pathway acts upstream of STAT3 phosphorylation, which inhibits tumorigenesis ^37, 38^. Meanwhile, ERK1/2 signals inhibit phosphorylation of STAT3, which could sensitize tumor cells to apoptosis.^39, 40^ STAT3 is a crucial protein in DLBCL whose phosphorylation can regulate multiple genes downstream that are associated with apoptosis and the cell cycle, such as Bcl-2, Bcl-xL and c-Myc.^41, 42, 43^ In addition, studies have shown that stimulation of the STAT3 pathway leads to poor overall survival and progression-free survival in DLBCL patients, in association with advanced stage and multiple extranodal sites of involvement.^44^ Accordingly, we explored whether DCZ3301 has an effect on the STAT3 signaling pathway. As expected, DCZ3301 treatment inactivated the STAT3 pathway to a significant extent in DLBCL cells. Furthermore, knockdown of the STAT3 gene in DLBCL enhanced the efficacy of DCZ3301, indicating that decreased STAT3 expression has a key role in DCZ3301-induced cytotoxicity. Overall, these results demonstrate that DCZ3301 induces apoptosis in DLBCL cells by decreasing STAT3 activation.

However, the upstream protein that induces STAT3 phosphorylation affected by DCZ3301 has not been explored. STAT3 is a substrate of tyrosine kinase Lyn.^16^ Lyn-phosphorylation-associated BCR activation also serves as an important part in the cell proliferation and apoptosis in lymphoma cells.^45, 46^ It has been reported that the small-molecule inhibitor to Lyn is a novel target for DLBCL patients.^47, 48^ Our results showed DCZ3301 inhibited Lyn activation along with decreased STAT3 activation. More importantly, the protein expressing of phospho-STAT3 in Lyn-OE cells suggests that DCZ3301 induces STAT3 phosphorylation by inhibiting Lyn activation directly in DLBCL.

In view of the finding that DCZ3301 exerts cytotoxic effects and apoptosis *in vitro*, we further examined its effects in a DLBCL xenograft mouse model. Specifically, mice were divided randomly into control and DCZ3301 treatment groups. Intravenous administration of 30 mg/kg DCZ3301 inhibited tumor growth significantly while inducing no obvious toxicity. DCZ3301 was well tolerated *in vivo*, and no significant differences in the weights of mice from the two groups were observed. Additionally, the phosphorylation of STAT3 was decreased by DCZ3301 treatment *in vivo* that verifies our previous result. Taken together, these results support the efficacy of DCZ3301 as a potent antitumor agent for DLBCL, both *in vitro* and *in vivo*.

Next, we assessed whether DCZ3301 can be used cooperatively with other chemotherapeutic drugs to treat DLBCL. Co-treatment with DCZ3301 and panobinostat synergistically induced further cell death of cancer cells. Panobinostat, an inhibitor that promotes the acetylation of histone and tubulin, inducing cancer cell apoptosis, has been shown to successfully treat multiple myeloma and approved by FDA.^49^ An earlier phase II study reported that panobinostat alone induces a consistent response in a proportion of relapsed DLBCL patients.^50^ Hence, combination of DCZ3301 with panobinostat may have potential as an effective therapeutic regimen to relieve symptoms in DLBCL patients. The mechanisms underlying the synergistic effects of DCZ3301 and panobinostat and whether these findings can be replicated *in vivo* remain to be established.

In summary, we have demonstrated a cytotoxic effect of DCZ3301 on human DLBCL cancer cells *in vitro*. DCZ3301 induced cell apoptosis and cell cycle arrest at the G2/M stage by regulating Akt, ERK1/2 and especially STAT3 pathways, which is triggered by Lyn phosphorylation. Synergistic effects on DLBCL cells were exerted by a combination of DCZ3301 and panobinostat. Consistent with *in vitro* results, DCZ3301 inhibited tumor growth *in vivo* with decreased phospho-STAT3. Our collective findings suggest that DCZ3301 may be applied as a potential therapy to improve outcomes in DLBCL patients. However, the detailed mechanisms and clinical effects of DCZ3301 in DLBCL require further research.

## Materials and methods

### Cell culture

The human cell lines, OCI-LY8 and NU-DUL-1, were a kind gift from Professor Xiaoyan Zhou from the Department of Pathology of Fudan University Shanghai Cancer Center (Shanghai, China). DB and SUDHL-4 cell lines were purchased from American Type Culture Collection (ATCC) (Mananssas, VA, USA). The TMD8 cell line was acquired from Professor Dongsheng Xu (Shanghai Tenth People’s Hospital, Tongji University of Medicine, Shanghai, China). Human OCI-LY8 cells were cultured in Iscove’s Modified Dulbecco’s Medium (Gibco, Life Technologies, Carlsbad, CA, USA) supplemented with 10% fetal bovine serum (FBS, Gibco) and 1% penicillin–streptomycin (Gibco), while all other cell lines were cultured in RPMI 1640 (Gibco) containing 10% FBS and 1% penicillin–streptomycin. All cells were incubated in a humidified atmosphere at 37 °C in 5% CO_2_. Normal PBMCs isolated from human peripheral blood provided voluntarily using Lymphoprep (Stemcell Technologies, Vancouver, Canada) were cultured in RPMI 1640 containing 10% FBS. Informed consent was obtained from each volunteer. These studies were approved by the institutional review board of Shanghai Tenth People’s Hospital (Shanghai, China).

### Reagents

A 16 mM DCZ3301 stock solution was dissolved in DMSO (Sigma, St. Louis, MO, USA) and stored at −20 °C. Antibodies for ERK1/2, phospho-ERK1/2, Akt, phospho-Akt, STAT3, phospho-STAT3, Lyn, phospho-Lyn, Syk, phospho-Syk, JAK2, phospho-JAK2, cleaved Caspase-8, Bax, B cell lymphoma-2 (Bcl-2), Bcl-xl, PARP, c-Myc, p21 and Actin (for western blotting) were purchased from Cell Signaling Technology (Danvers, MA, USA). Caspase-9, cleaved caspase-3, p-CHK2, cdc25A, cdc25C and cyclinB1 antibodies were purchased from Abcam (Cambridge, MA, USA). IL-6 and IGF-1 (R&D Systems, Minneapolis, MN, USA) dissolved in phosphate-buffered saline (PBS) containing 0.1% albumin from bovine serum albumin (BSA) were prepared as 10 *μ*g/ml stock solution and stored at −20 °C. Panobinostat and vorinostat were provided by Selleck Chemicals (Houston, TX, USA). CCK-8 was obtained from Yeasen (Shanghai, China), the Annexin-V/PI apoptosis detection kit from BD Pharmingen (Franklin Lakes, NJ, USA) and the JC-1 Mitochondrial Membrane Potential Detection Kit from Beyotime Institute of Biotechnology (Shanghai, China).

### Cell proliferation assay

DLBCL cells were seeded at a density of 2 × 10^5^ cells/ml, treated with specific concentrations of DCZ3301 and plated into 96-well plates for 48 h in the incubator. At the end of the incubation period, 10 *μ*L CCK-8 solution was added to each well and the plates returned to the incubator for an additional 2 h at 37 °C. Absorbance was measured at 450 nm using a microplate reader.

### Cell apoptosis assay

Cell apoptosis was measured using the Annexin-V/PI Apoptosis Detection Kit. OCI-LY8 and NU-DUL-1 cells were seeded at a density of 2 × 10^5^ cells/ml into 12-well plates treated with DCZ3301 and incubated for 24, 48 or 72 h. Normal PBMCs were treated with DCZ3301 for 48 h. According to the manufacturer’s instructions, cells were stained with the Annexin-V/PI dye protected from light and analyzed using a BD FASC Canto II flow cytometer (BD). Apoptotic cells were identified as both Annexin-V+/PI− (early apoptosis) and Annexin-V+/PI+ (late apoptosis).

### Cell cycling assay

DLBCL cells were cultured in serum-free medium for 12 h and subsequently seeded at a density of 2 × 10^5^ cells/ml into 12-well culture plates treated with DCZ3301 for 8, 12 or 24 h. The collected cells were washed with precooled PBS and fixed with 75% ethanol at −20 °C overnight. Ethanol-fixed cells were washed with PBS, stained with 500 *μ*L PI (BD) for 15 min at room temperature and assessed on a flow cytometer.

### MMP assay

Changes in MMP resulting in cell apoptosis were detected via flow cytometry using a JC-1 Kit. DLBCL cells were treated with DCZ3301 for 24 or 48 h and stained with 2 *μ*M JC-1 followed by incubation at 37 °C for 15 to 30 min, according to the manufacturer’s instructions. Cells were subsequently washed with PBS and analyzed with a flow cytometer.

### Western blotting assay

Cells treated with different concentrations of DCZ3301 were lysed in lysis buffer (100 mM Tris–HCl, pH 6.8, 4% SDS, 20% glycerol). Cytosolic proteins (30 *μ*g per lane) were electrophoretically separated on a 10% or 12.5% sodium dodecyl sulfate-polyacrylamide gel and transferred to polyvinylidene difluoride or nitrocellulose membranes, blocked in 5% defatted milk or 5% BSA for 1 h and incubated overnight at 4 °C with the relevant primary antibodies. Membranes were washed with Tween-20-PBS (PBST) three times and incubated with the corresponding secondary antibodies (anti-rabbit or anti-mouse IgG) for 1 h at room temperature. Protein signals were subsequently detected using the Odyssey two-color infrared laser imaging system (LI-COR, Lincoln, NE, USA).

### Silencing of STAT3 in DLBCL cell lines

siRNA oligonucleotides against *STAT3* (CCGTGGAACCATACACAAA) and negative control siRNA (Sigma) were transfected into OCI-LY8 and NU-DUL-1 cells cultured in Opti-MEM (Gibco) by means of Lipofectamine RNAiMAX transfection reagent (Invitrogen, Carlsbad, CA, USA) up to a final concentration of 100 nM. Cells were incubated for 24 h and collected after adding culture medium containing 20% FBS into starved cells.

### Overexpression of Lyn in DLBCL cell lines

We overexpressed oncogene Lyn in OCI-LY8 and NU-DIL-1 cell lines by transfecting a plasmid carrying the sequence of Lyn. We cloned the CDDS sequence of LYN isoform A (P07948) and connected with the empty vector (pCDH1-CMV-MCS-EF1-RFP cDNA cloning and expression vector), which is a kind gift from Dr. Junhua Z. The recombination plasmid was confirmed by cDNA sequencing that is exactly 1539 bp. The plasmid was transfected into OCI-LY8 and NU-DUL-1 cells as the overexpression (OE) group while the negative control group was transfected with an empty vector via Lipofectamine 3000 transfection reagent (Invitrogen) for 48 h.

### Tumor xenograft model

Six-week-old male nude mice (athymic, BALB/c nu/nu) were purchased from Shanghai Laboratory Animal Center (SLAC, Shanghai, China), housed in a standard animal laboratory and fed a standard diet with free access to water. Human DLBCL OCI-LY8 cells (2 × 10^6^) were suspended in 100 *μ*l serum-free culture medium and subcutaneously injected into the upper flank region of nude mice. After tumor size had reached an approximate volume of 100 mm^3^, six mice were randomly divided into control (5% DMSO and saline only) and DCZ3301-treated groups (40 mg/kg DCZ3301 in 5% DMSO and saline) (*n*=3/group). Mice were administered DCZ3301 via intraperitoneal injection (40 mg/kg/day) for 12 days and assessed for tumor size and weight each day. At the end of the experimental period, all mice were killed, and the tumors were obtained and imaged. Tumors of mice were embedded in paraffin after imaging, and H&E, TUNEL and anti-phospho-STAT3 antibody staining of 5-*μ*m-thick tissue sections of tumors and livers was performed. All animal-related procedures were approved by the Animal Care and Use Committee of The Tenth People’s Hospital (Shanghai, Tongji University). This research was approved by the Science and Technology Commission of Shanghai Municipality (ID: SYXK 2011-0111).

### Statistical analysis

Data are expressed as means±S.D. Comparisons among the experimental groups were conducted with Student’s *t*-test, and the significance of multiple comparisons was determined using one-way ANOVA. All statistical analyses were performed with the SPSS v20.0 statistical analysis software (IBM Corp., Armonk, NY, USA). A *P*-value of ≤0.05 was considered significant.
